# Endogenous circadian time genes expressions in the liver of mice under constant darkness

**DOI:** 10.1186/s12864-020-6639-4

**Published:** 2020-03-12

**Authors:** Huan Li, Shiyao Zhang, Wenxiang Zhang, Siyu Chen, Anjara Rabearivony, Yujie Shi, Jie Liu, Christopher J. Corton, Chang Liu

**Affiliations:** 1 School of Life Sciences and Technology, China Pharmaceutical University, Nanjing, China; 2 Key Laboratory of Basic Pharmacology of Ministry of Education, Zunyi Medical University, Zunyi, Guizhou China; 3 Computational Toxicology Division, US Environmental Protection Agency, Research Triangle Park, NC USA

**Keywords:** Constant darkness, Circadian time, RNA sequence, MetaCycle, Mouse liver, RT-qPCR

## Abstract

**Background:**

The circadian rhythms regulate physiological functions and metabolism. Circadian Time (CT) is a unit to quantify the rhythm of endogenous circadian clock, independent of light influence. To understand the gene expression changes throughout CT, C57BL/6 J mice were maintained under constant darkness (DD) for 6 weeks, and the liver samples were collected starting at 9:00 AM (CT1), and every 4 h in a 24-h cycle (CT5, CT9, CT13, CT17 and CT21). Total RNA was extracted and subjected to RNA-Seq data (deposited as GSE 133342, L-DD). To compare gene oscillation pattern under normal light-dark condition (LD, GSE114400) and short time (2 days) dark-dark condition (S-DD, GSE70497), these data were retried from GEO database, and the trimmed mean of M-values normalization was used to normalize the three RNA-seq data followed by MetaCycle analysis.

**Results:**

Approximate 12.1% of the genes under L-DD exhibited significant rhythmically expression. The top 5 biological processes enriched in L-DD oscillation genes were mRNA processing, aromatic compound catabolic process, mitochondrion organization, heterocycle catabolic process and cellular nitrogen compound mitotic catabolic process. The endogenous circadian rhythms of clock genes, P450 genes and lipid metabolism genes under L-DD were further compared with LD and S-DD. The oscillation patterns were similar but the period and amplitude of those oscillation genes were slightly altered. RT-qPCR confirmed the selected RNA sequence findings.

**Conclusions:**

This is the first study to profile oscillation gene expressions under L-DD. Our data indicate that clock genes, P450 genes and lipid metabolism genes expressed rhythmically under L-DD. Light was not the necessary factor for persisting circadian rhythm but influenced the period and amplitude of oscillation genes.

## Background

Organisms adapt and respond optimally to the 24-h light-dark cycles induced by the earth autorotation, and such an adaptation is known as the circadian clock which orchestrates and maintains a 24-h rhythm of mammalian daily activities [1, 2]. The circadian rhythm is driven by circadian clock genes, which are under control of a hierarchical timing system consisting of the master circadian clock located in the hypothalamic suprachiasmatic nucleus (SCN) and a set of peripheral clocks located at the liver and peripheral organs [3, 4]. The master circadian clock relays temporal information to peripheral clocks through autonomic innervation, glucocorticoids, body temperature and feeding, while peripheral clocks are also influenced by the local metabolic status of the tissues in which they reside [3, 5]. The core clock is driven by the transcriptional activators including *Clock*, *Bmal1* (*Arnt1*) and *Npas2*, which heterodimerize (CLOCK/BMAL1, NPAS2/BMAL1) and bind to DNA sequences in the promoters of regulated genes called E-box response elements [5, 6]. BMAL1/CLOCK drives the transcription of several distinct negative feedback genes, including two Cryptochrome (*Cry1*, *Cry2*) genes and three Period genes (*Per1*, *Per2, Per3*), whose products multimerize and suppress the activation of the CLOCK:BMAL1 complex. The CLOCK/BMAL1 heterodimer also robustly governs the circadian expression of nuclear orphan receptors *Rev-erb*α (*Nr1d1*), the PAR-bZip family members *Dbp* and other clock targeted genes [6, 7].

The circadian clock, as a rhythmic epigenomic programmer, controls a group of metabolic processes, such as the sleep/wake cycle, the fasting/feeding cycle, glucose homeostasis, as well as lipid and bile acid metabolism [2, 8]. The crosstalk between circadian clock and metabolism is known and the disorder of circadian clock destroys metabolic homeostasis [8–11]. For example, *Clock* mutant mice developed hyperglycemia, hypo-insulinemia, and obesity [12]. Specific knockout of *clock* gene in liver resulted in hyperglycemia [13]. Deletion of the *Rev-erbα* gene decreased bile acid synthesis and reduced bile acid accumulation in the liver [14]. In addition, the rate-limiting enzymes of some metabolic processes such as *Cyp7a1* were regulated by circadian clock genes [9]. Taken together, the crosstalk between circadian clock and metabolism is vital in maintaining metabolic homeostasis [10].

Circadian rhythms are generated by intrinsic oscillation of their specific central clock [15]. Light is a conspicuous zeitgeber for the circadian system (Zeitgeber is defined as a rhythmically occurring phenomenon acting as a cue in the regulation of body’s circadian rhythms). However, under the constant light or dark conditions, a rhythm still persists [16]. Three models are often used to perform circadian researches: light-dark conditions (12 h light:12 h dark, LD), constant darkness (DD), and constant light (LL) [17, 18]. Although most clock researches are carried out using the LD model, several studies described circadian rhythm under the DD condition where the time is referred as CT units, different from Zeitgeber Time (ZT) [17, 19–21].

In the DD model, animals are shielded from light to eliminate entrainment effects on the circadian clock, known as “free running rhythm”, which is regarded as mammalian biological signal in metabolism [18, 22]. Under DD condition, wheel-running experiment was performed to monitor the rhythm of locomotor activity [23]. It has been reported light and darkness had acute effects on the activity and temperature rhythms of a subterranean rodent, the Anillaco tuco-tuco [24]. Besides, aberrant emotional behaviors and cognition were related to altered light conditions [25]. The composition of the murine gut microbiome, memory Function, and plasma metabolome was influenced by DD [26]. Under the DD model and fast-refeeding conditions, we have identified the *Angptl8* as a hepatokine that mediates food-driven resetting of hepatic clock and diurnal rhythms of metabolic genes in mice [27]. Therefore, DD is an important factor not only for circadian clock but also for metabolism. To examine the circadian rhythm of circadian clock genes, P450 enzyme genes, and lipid metabolism genes under DD condition would benefit and impact the metabolism research.

The present study was conducted in mice acclimated to DD conditions for 6 weeks (called long time dark-dark condition, L-DD) to eliminate light influence. The liver samples were collected based on CT time points and subjected to RNA sequencing to profile the changes of gene expression abundance in the liver [27]. MetaCycle was used to screen the oscillation genes and real-time RT-qPCR was used to confirm the gene expression patterns. To further understand the endogenous rhythm of oscillation genes in metabolism, comparisons of P450 enzyme genes, lipid metabolism genes, and clock genes under LD, L-DD and shorter (2 days) constant darkness (S-DD) were made. Our results clearly demonstrate that the endogenous circadian rhythm of clock genes, P450 genes and lipid metabolism genes robustly persists under L-DD. The oscillation patterns were similar but the period and amplitude of those oscillation genes were slightly altered. Light was not the necessary factor for persisting circadian rhythm but influenced the period and amplitude of oscillation genes.

## Results

**Fig. 1:**
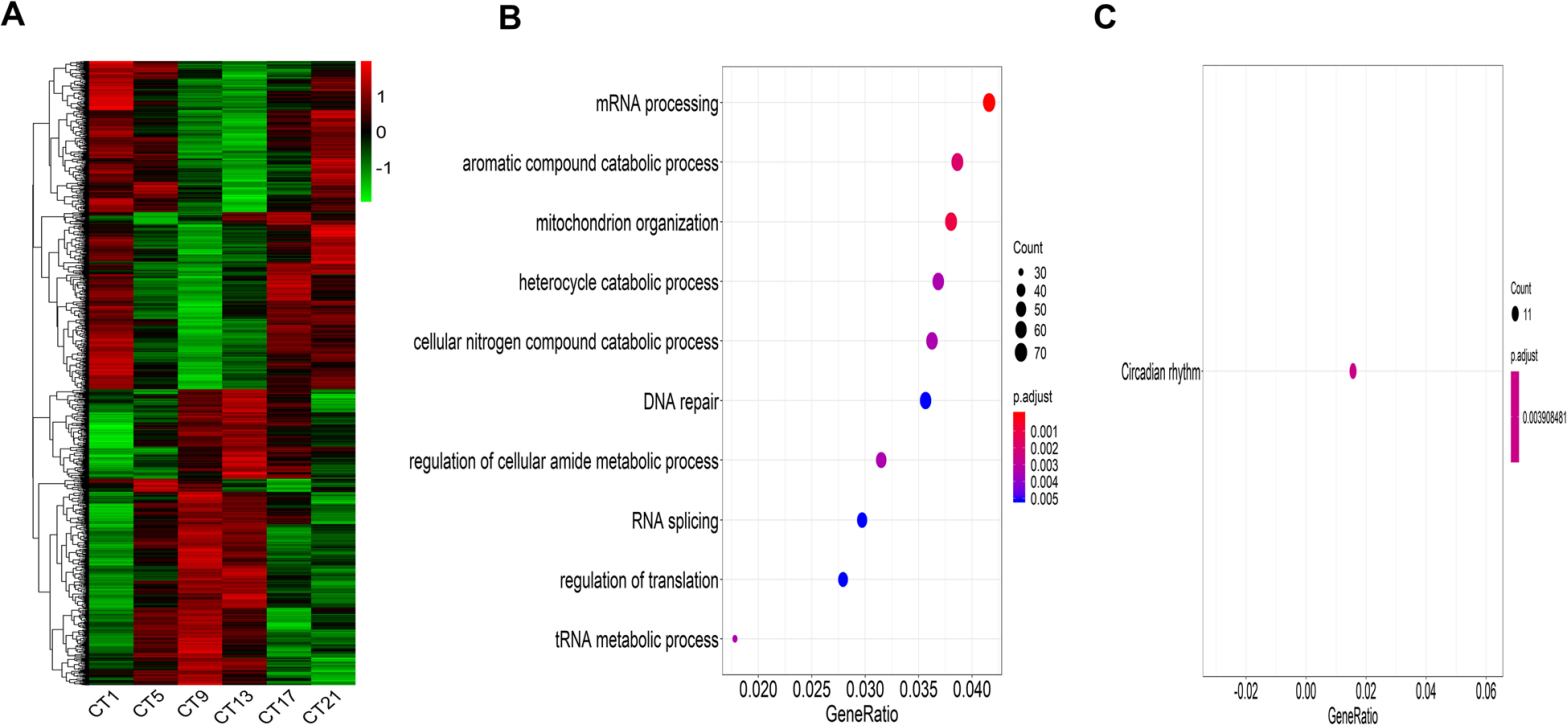
Oscillation genes profile and biological function analysis under L-DD. RNA-Seq data under L-DD was normalized by TMM and subjected to MetaCycle to screen the oscillation genes. GO (Biological process) and KEGG were performed to analyze the biological function of those oscillation genes. **a** Heatmap displaying oscillation genes under L-DD. **b** GO analysis of oscillation genes under L-DD. **c** KEGG analysis of oscillation genes under L-DD

The RNA-sequencing data of L-DD was first trimmed mean of M-values (TMM) normalized, followed by MetaCycle analysis to identify oscillation genes. The results showed that 12.1% of the genes under L-DD exhibited a statistically significant (*P* < 0.05) rhythmically expression (Additional file 1). These oscillation genes were visualized in the heatmap (Fig. 1a). To further understand the functional and biological pathways of those oscillation genes, Gene ontology (GO) and Kyoto encyclopedia of genes and Genomes (KEGG) analysis were performed. For the GO (biological processes) analysis, the top 5 enriched numbers of L-DD oscillation genes were mRNA processing, aromatic compound catabolic process, mitochondrion organization, heterocycle catabolic process and cellular nitrogen compound mitotic catabolic process (Fig. 1b). The KEGG analysis showed that the oscillation genes under L-DD were involved in the circadian rhythm (Fig. 1c).

**Fig. 2:**
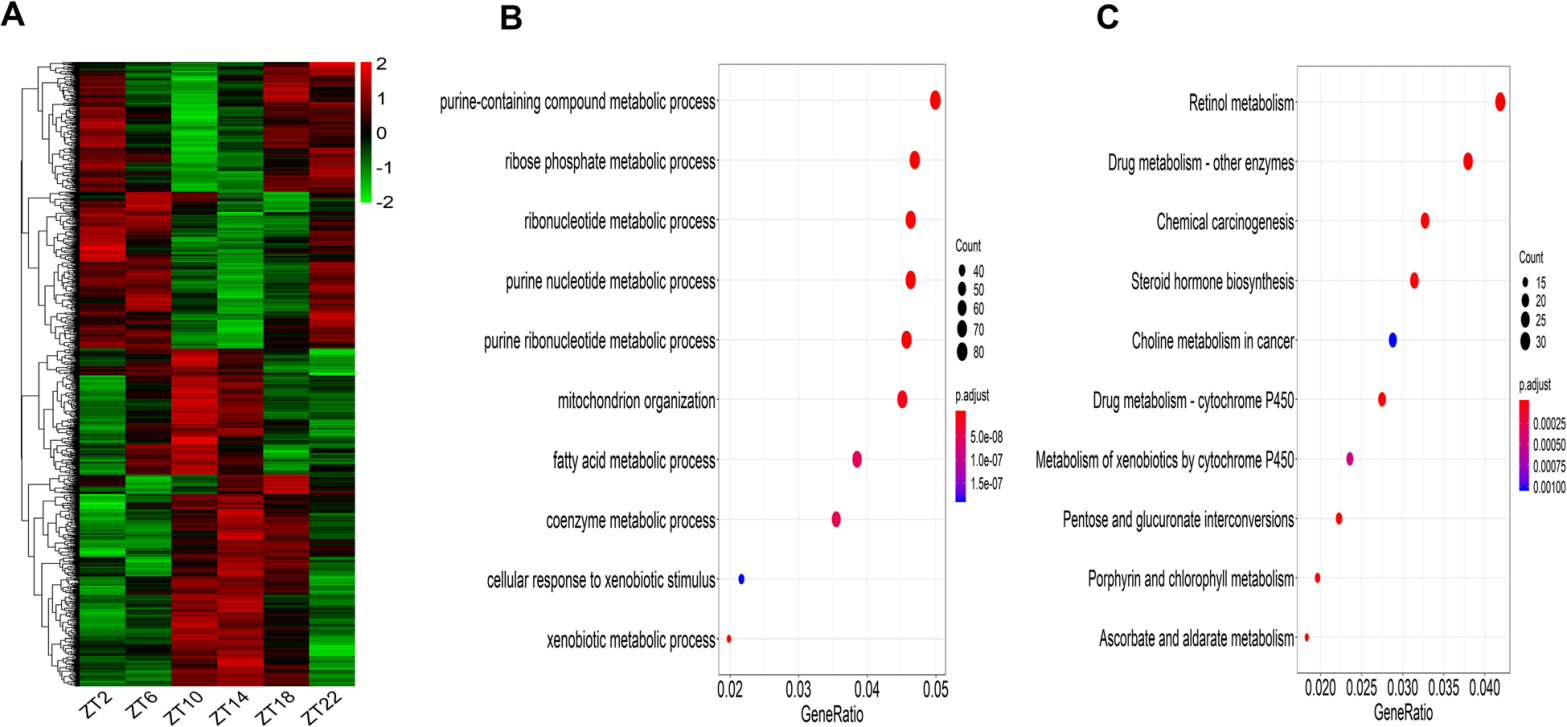
Oscillation genes profile and biological function analysis under LD. RNA-Seq data under LD was normalized by TMM and subjected to MetaCycle to screen the oscillation genes. GO (Biological process) and KEGG were performed to analyze the biological function of those oscillation genes. **a** Heatmap displaying oscillation genes under LD. **b** GO analysis of oscillation genes under LD. **c** KEGG analysis of oscillation genes under LD

**Fig. 3:**
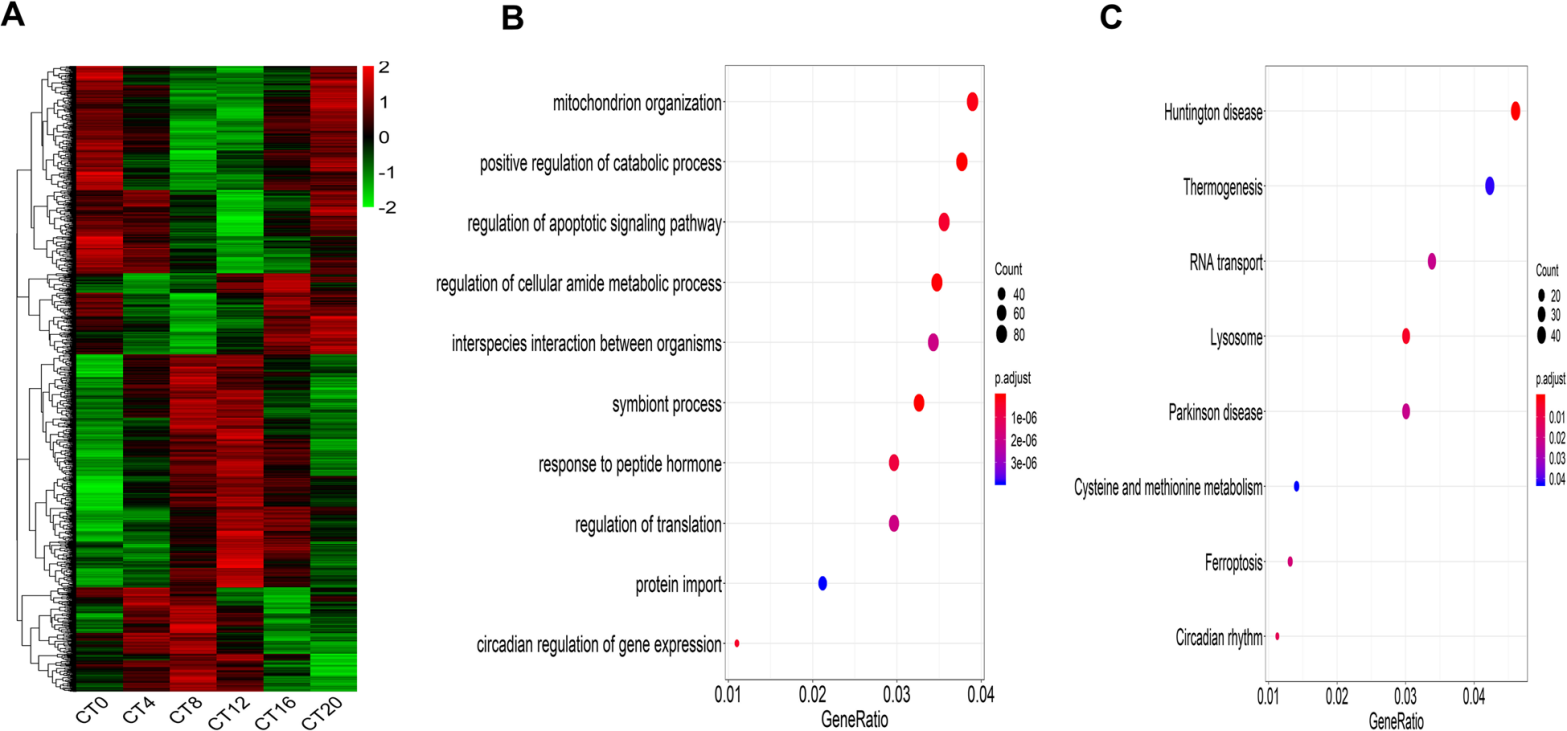
Oscillation genes profile and biological function analysis under S-DD. RNA-Seq data under S-DD was normalized by TMM and subjected to MetaCycle to screen the oscillation genes. GO (Biological process) and KEGG were performed to analyze the biological function of those oscillation genes. **a** Heatmap displaying oscillation genes under S-DD. **b** GO analysis of oscillation genes under S-DD. **c** KEGG analysis of oscillation genes under S-DD

In order to compare the gene oscillation patterns under L-DD in the present study, with those under LD and S-DD, the RNA-sequence data of LD (GSE114400) [28] and S-DD (GSE70947) [29] were retrieved from the GEO database and subjected to the MetaCycle analysis after the same TMM normalization. The raw filtered results were listed in additional file 2 and additional file 3, respectively. The oscillation gene patterns of LD were shown in Fig. 2a. GO analysis showed the oscillation genes under LD were enriched in the ribonucleotide metabolic process, mitochondrion organization and fatty acid metabolic process (Fig. 2b). KEGG analysis revealed that those oscillation genes were enriched on retinol metabolism, steroid hormone biosynthesis and drug metabolism-cytochrome P450 pathway (Fig. 2c). The oscillation gene patterns under S-DD were shown in Fig. 3a, The oscillation genes under S-DD were mainly gathered at mitochondrion organization, catabolic process, apoptotic signaling pathway and cellular amide metabolic process with GO analysis (Fig. 3b). KEGG analysis illustrated those oscillation genes participated in Huntington disease, Thermogenesis, RNA transport and Lysosome pathway (Fig. 3c). It is clear that under L-DD conditions, GO was mainly enriched in catabolic metabolism, and KEGG was enriched in circadian rhythm, which was quite different from LD, and somewhat different from S-DD.

**Fig. 4:**
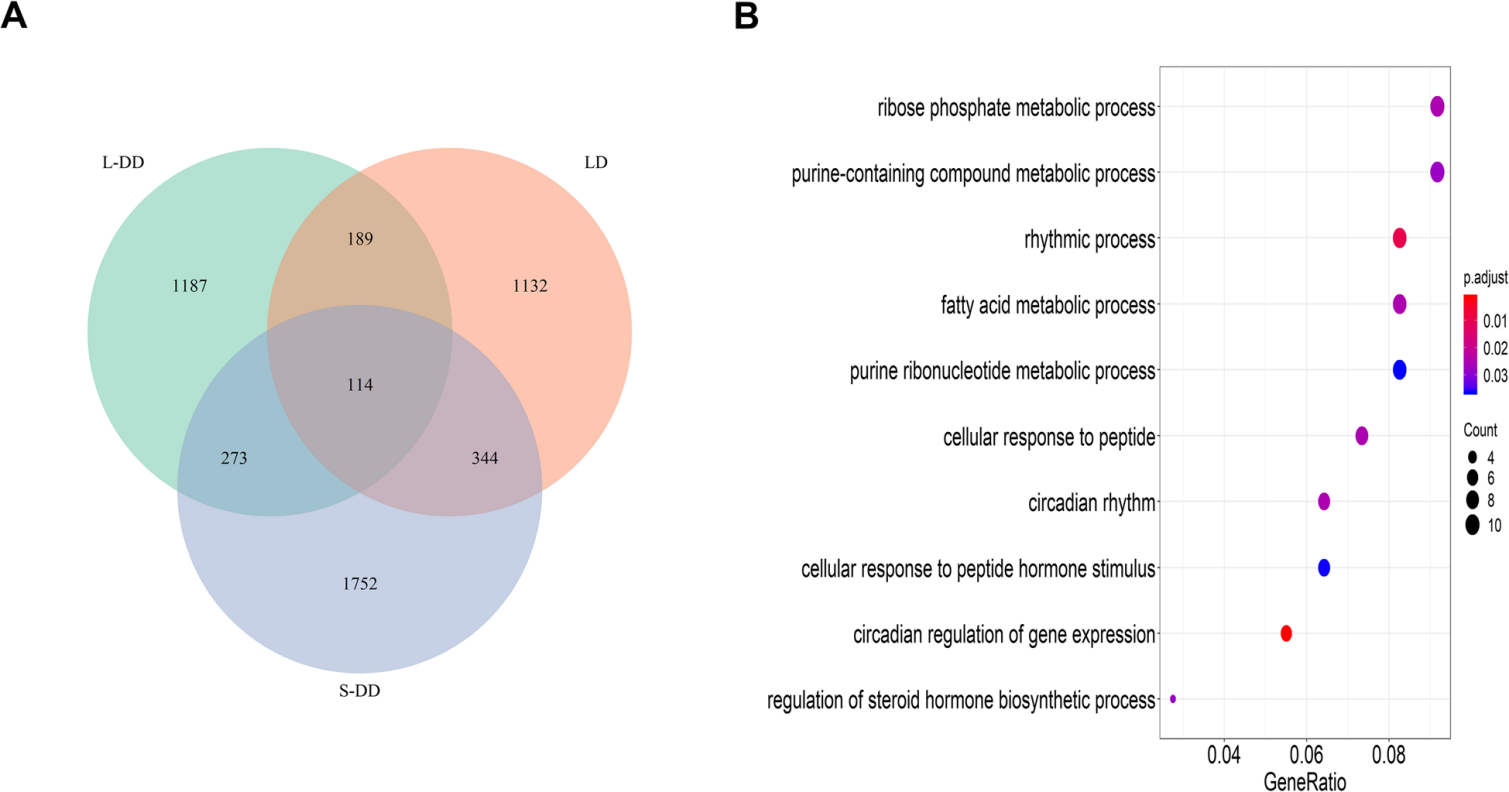
Comparisons of hepatic oscillation genes under LD, S-DD and L-DD. **a** Venn diagram displaying the overlapping number of oscillation genes under LD (red), S-DD (green) and L-DD (blue). **b** KEGG analysis of overlapping genes under three conditions

To further study the characteristics of oscillation genes under L-DD, the oscillation genes under three different light conditions of LD, S-DD and L-DD were compared. As shown in Venn diagram (Fig. 4a), there are 1779 hepatic oscillation genes under LD condition (1779/14498*100 = 12.3%), 2483 under S-DD condition (2483/13522*100 = 17.9%) and 1763 under L-DD condition (1763/14518*100 = 12.1%). Comparison of the oscillation genes between LD and S-DD showed that 458 genes expressed rhythmically both at LD and S-DD. Three hundred eighty-seven genes oscillated at both S-DD and L-DD. Three hundred three genes oscillated at both L-DD and LD. Notably, 114 genes persisted oscillating at all the three conditions, suggesting their oscillations were endogenous. GO analysis revealed that the 114 genes were involved in ribose phosphate metabolic process, purine-containing compound metabolic process, rhythmic process, fatty acid metabolic process and purine ribonucleotide metabolic process (Fig. 4b).

**Fig. 5:**
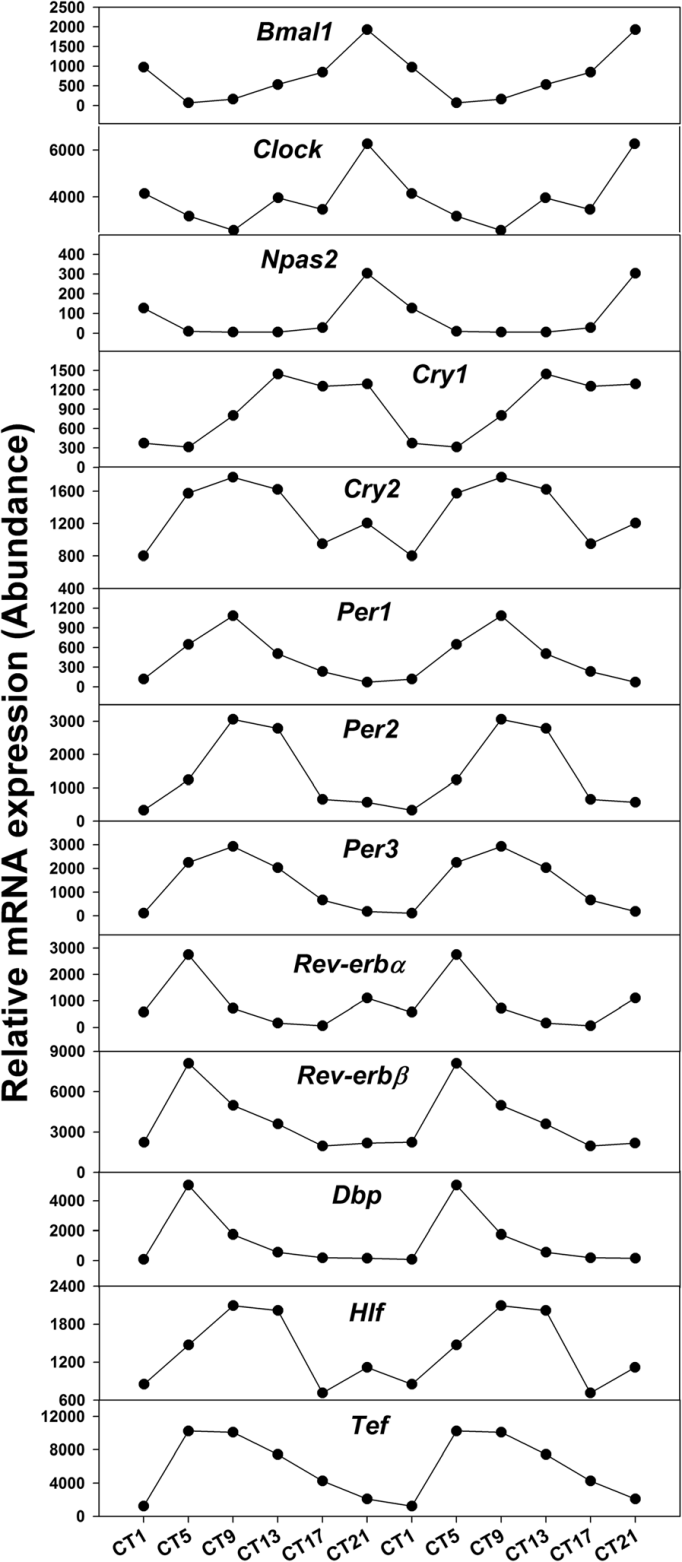
Oscillation patterns of hepatic circadian clock genes in the L-DD model. Mice were acclimated to constant dark for 6 weeks, and livers were harvested at 9:00 am (CT1), and then every 4 h (CT5, CT9, CT13, CT17, and CT21). RNA-Seq data was presented as reads count of pooled samples

To better understand the oscillation pattern under L-DD, Fig. 5 showed that the circadian clock genes exhibited typical oscillation patterns. Table 1 listed the period and amplitude of circadian clock genes under LD, S-DD and L-DD. Compared to LD and S-DD conditions, the periods of clock core genes *Bmal1*, *Clock* and *Npas2* got longer but the amplitudes were slightly decreased. For period genes *Per1* and *Per2*, L-DD significantly increased their period but did not influence the period of *Per3*. The period of *Cry1* gene got longer under L-DD but the amplitude was unaltered. For *Cry2* gene, its period became shorter but the amplitude was increased under L-DD. L-DD had no influence on period of *Rev-erbα* but increased its amplitude, on the contrary, increased period of *Rev-erbβ* was observed under L-DD. For clock-targeted genes, compared to LD condition, the period of *Dbp* decreased but the period of *Tef* increased. The amplitudes of *Dbp*, *Tef* and *Hlf* all increased under L-DD.

**Table 1:** Comparisons of hepatic circadian clock genes under LD, S-DD and L-DD conditions

Name	***P***-value			Period (hour)			_rAmplitude		
	LD	S-DD	L-DD	LD	S-DD	L-DD	LD	S-DD	L-DD
Bmal1	0.07	0.00	0.28	21.56	22.06	22.47	0.51	0.68	0.50
Clock	0.01	0.00	0.48	20.33	20.93	23.25	0.08	0.13	0.06
Npas2	0.02	0.00	0.01	22.06	21.14	24.45	2.25	3.86	2.50
Per1	0.05	0.00	0.01	21.00	22.56	24.96	0.62	0.54	0.95
Per2	0.08	0.01	0.00	20.16	22.95	21.03	0.39	0.42	0.40
per3	0.01	0.00	0.03	22.73	23.4	22.86	0.29	0.47	0.72
Cry1	0.17	0.02	0.04	21.59	23.05	23.31	0.28	0.30	0.26
Cry2	0.22	0.14	0.04	25.54	24.78	20.05	0.08	0.06	0.15
Rev-erbα	0.17	0.04	0.15	21.97	22.55	21.33	0.64	0.65	1.07
Rev-erbβ	0.01	0.00	0.08	20.19	22.41	22.62	0.16	0.26	0.19
Dbp	0.02	0.00	0.24	22.11	20.90	20.61	0.46	0.95	0.85
Tef	0.02	0.00	0.04	22.73	21.70	24.10	0.13	0.19	0.28
Hlf	0.04	0.02	0.02	20.12	22.34	20.02	0.11	0.06	0.19

Note: 0.00 indicates that the values are less than 0.004

**Fig. 6:**
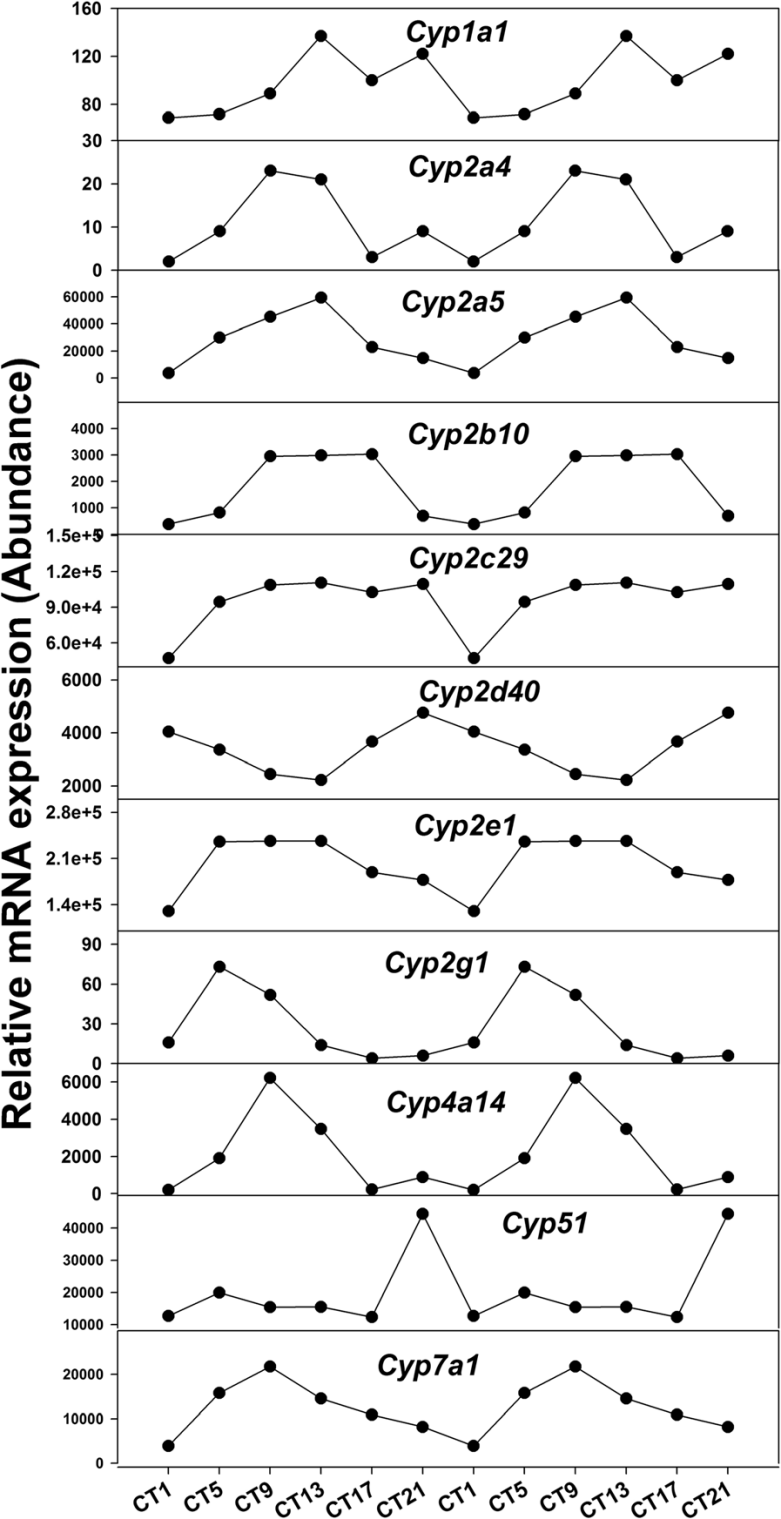
Oscillation patterns of hepatic cytochrome P450 genes in the L-DD model. Mice were acclimated to constant dark for 6 weeks, and livers were harvested at 9:00 am (CT1), and then every 4 h (CT5, CT9, CT13, CT17, and CT21). RNA-Seq data was presented as reads count of pooled samples

Cytochrome P450 (CYP450) enzymes are the key enzymes in the liver involved in the metabolism of drugs, steroids, vitamins, and other chemicals [30]. CYP450 superfamily is typical to represent most common phase I drug-metabolizing enzymes [31]. For example, CYP1-CYP3 members in the CYP450 families are responsible for the phase I-dependent metabolism of 70–80% clinically used drugs [32]. In addition, our previous study has reported that some of the CYP450 genes were rhythmically expressed in the mouse liver under LD condition [33]. The presented study illustrated that CYPs exhibited robust circadian rhythm in mouse liver under L-DD. Figure 6 showed the oscillation pattern of some P450 genes with typical circadian rhythm. Table 2 presented the periods and amplitudes of those oscillation CYPs genes under LD, S-DD and L-DD. Cytochrome P450 families 1–3 (CYP1-CYP3) are involved in drug and steroid metabolism [30]. Compared to the LD and S-DD, the periods of *Cyp1a1*, *Cyp2a4* and *Cyp2d40* decreased but for *Cyp2a5*, *Cyp2b10* and *Cyp2c29*, their periods were increased under L-DD. L-DD extended the period of *Cyp2e1* but had no influence on the period of *Cyp2g1*. *Cyp4a14* is responsible for fatty acid metabolism, and its period was downregulated under L-DD. *Cyp7a1* is involved in bile acid biosynthesis [34], and *Cyp51* is an essential enzyme in sterol biosynthesis [35]. Periods of the two genes significantly increased under L-DD. Compared to LD and S-DD, L-DD failed to alter amplitudes of most gens including *Cyp2a5, Cyp2c29*, *Cyp2d40*, *Cyp2e1*, *Cyp51* and *Cyp7a1*. However, for *Cyp1a1, Cyp2g1* and *Cyp4a14*, their amplitudes were increased. The amplitudes of *Cyp2a4* and *Cyp2b10* were decreased under L-DD.

**Table 2:** Comparisons of hepatic cytochrome P450 genes under LD, S-DD and L-DD conditions

Name	***P***-value			Period (hour)			rAmplitude		
	LD	S-DD	L-DD	LD	S-DD	L-DD	LD	S-DD	L-DD
Cyp1a1	0.14	0.83	0.05	23.59	25.33	23.02	0.19	0.15	0.39
Cyp2a4	0.01	0.04	0.05	22.44	20.12	20.34	1.21	0.19	0.52
Cyp2a5	0.01	0.01	0.02	22.74	24.32	25.57	0.17	0.16	0.22
Cyp2b10	0.81	0.02	0.06	21.33	22.76	25.18	0.97	0.13	0.46
Cyp2c29	0.50	0.87	0.03	24.45	21.33	25.34	0.01	0.05	0.05
Cyp2d40	0.01	0.94	0.03	25.26	24.00	20.28	0.06	0.06	0.07
Cyp2e1	0.03	0.09	0.00	21.71	24.45	23.92	0.03	0.04	0.05
Cyp2g1	0.00	0.06	0.01	22.91	20.31	22.48	0.88	0.61	1.12
Cyp4a14	0.66	0.11	0.04	21.63	20.23	20.34	0.16	0.05	0.54
Cyp51	0.15	0.57	0.02	20.51	21.33	25.33	0.09	0.04	0.06
Cyp7a1	0.82	0.05	0.04	21.33	21.33	22.86	0.13	0.13	0.15

Note: 0.00 indicates that the values are less than 0.004

**Fig. 7:**
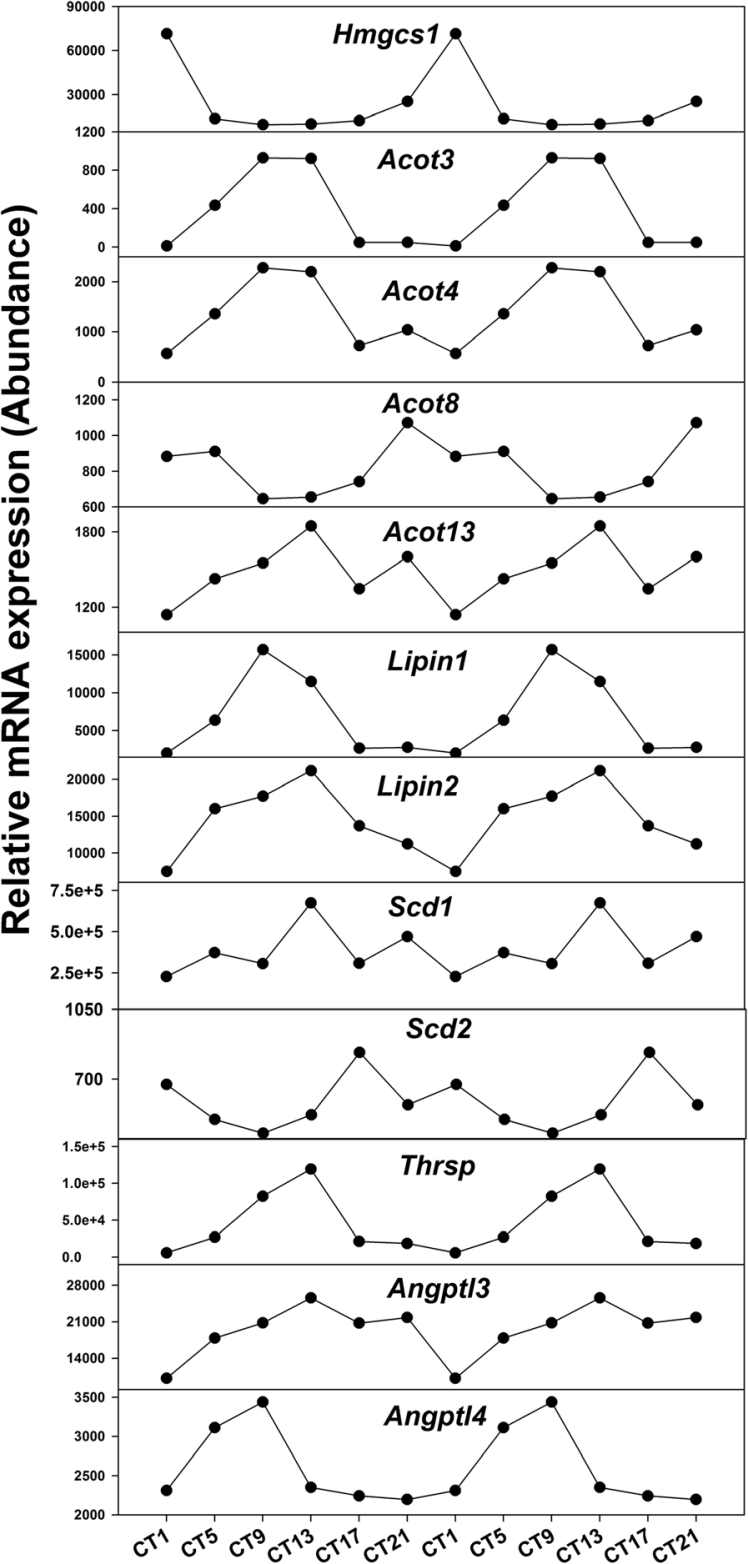
Oscillation patterns of hepatic lipid metabolism genes under the L-DD condition. Mice were acclimated to constant dark for 6 weeks, and livers were harvested at 9:00 am (CT1), and then every 4 h, (CT5, CT9, CT13, CT17, and CT21) for 6 time-points. RNA-Seq data was presented as reads count of pooled samples

The oscillation genes that regulate cholesterol, lipid and fatty acid metabolism are shown in Fig. 7 and Table 3. *Hmgcs1* encode the enzyme HMG-CoA (the substrate of *Hmgcr*) and play an important role in cholesterol metabolism [36]. Compared to LD, the period of *Hmgcs1* increased 5 h but its amplitude had no change under L-DD. Acyl-CoA thioesterase (Acot) genes act as auxiliary enzymes in the α- and β-oxidation of various lipids in peroxisomes [37]. Compared to LD, the period of *Acot3*, *Acot4*, *Acot8* slightly decreased under L-DD. For the amplitude, L-DD increased the amplitude of *Acot3*, and *Acot4* but had no influence on *Acot13* and *Acot8*. *Lipin1*, *Lipin2* and *Thrsp* are involved in lipogenesis [38]. Compared to LD, the period of *Thrsp* gradually decreased but amplitude increased under S-DD and L-DD. On the contrary, the periods of *Lipin1* and *Lipin2* were increased under L-DD. Stearoyl-Coenzyme A desaturase (Scd) genes encode the key enzymes involved in the conversion of saturated fatty acids into monounsaturated fatty acids [39]. L-DD decreased the period of *Scd1* and *Scd2*. The recent research illustrated *Angptl3* and *Angptl4* played important roles in lipid metabolism [40]. The presented study found that the expression of *Angptl3* and *Angptl4* showed circadian rhythm under LD and L-DD. Compared to LD, the period of *Angptl3* became long under L-DD.

**Table 3:** Comparisons of hepatic cholesterol and fatty acids metabolism genes under LD, S-DD and L-DD conditions

Name	***P***-value			Priod (hour)			rAmplitude		
	LD	S-DD	L-DD	LD	S-DD	L-DD	LD	S-DD	L-DD
Hmgcs1	0.05	NA	0.02	20.02	NA	25.02	0.19	NA	0.13
Acot3	0.00	0.01	0.01	22.19	20.05	20.69	1.07	0.20	1.77
Acot4	0.01	0.04	0.02	22.48	20.19	20.41	0.18	0.08	0.24
Acot8	0.35	0.19	0.02	21.87	22.89	20.37	0.04	0.03	0.06
Acot13	0.00	0.11	0.00	22.80	25.29	22.64	0.04	0.03	0.07
Lpin1	0.04	0.00	0.00	20.22	22.62	20.95	0.21	0.17	0.25
Lpin2	0.02	0.01	0.01	22.03	22.12	24.32	0.08	0.07	0.10
Scd1	0.40	0.05	0.60	25.33	23.57	22.15	0.01	0.02	0.03
Scd2	0.00	0.03	0.70	22.69	22.99	21.33	0.05	0.07	0.14
Thrsp	0.45	0.06	0.00	24.19	23.11	21.30	0.06	0.17	0.21
Angptl3	0.03	0.91	0.01	23.73	24.00	24.87	0.02	0.00	0.07
Angptl4	0.03	0.43	0.07	24.05	20.12	23.16	0.10	0.09	0.07

Note: 0.00 indicates that the values are less than 0.004

**Fig. 8:**
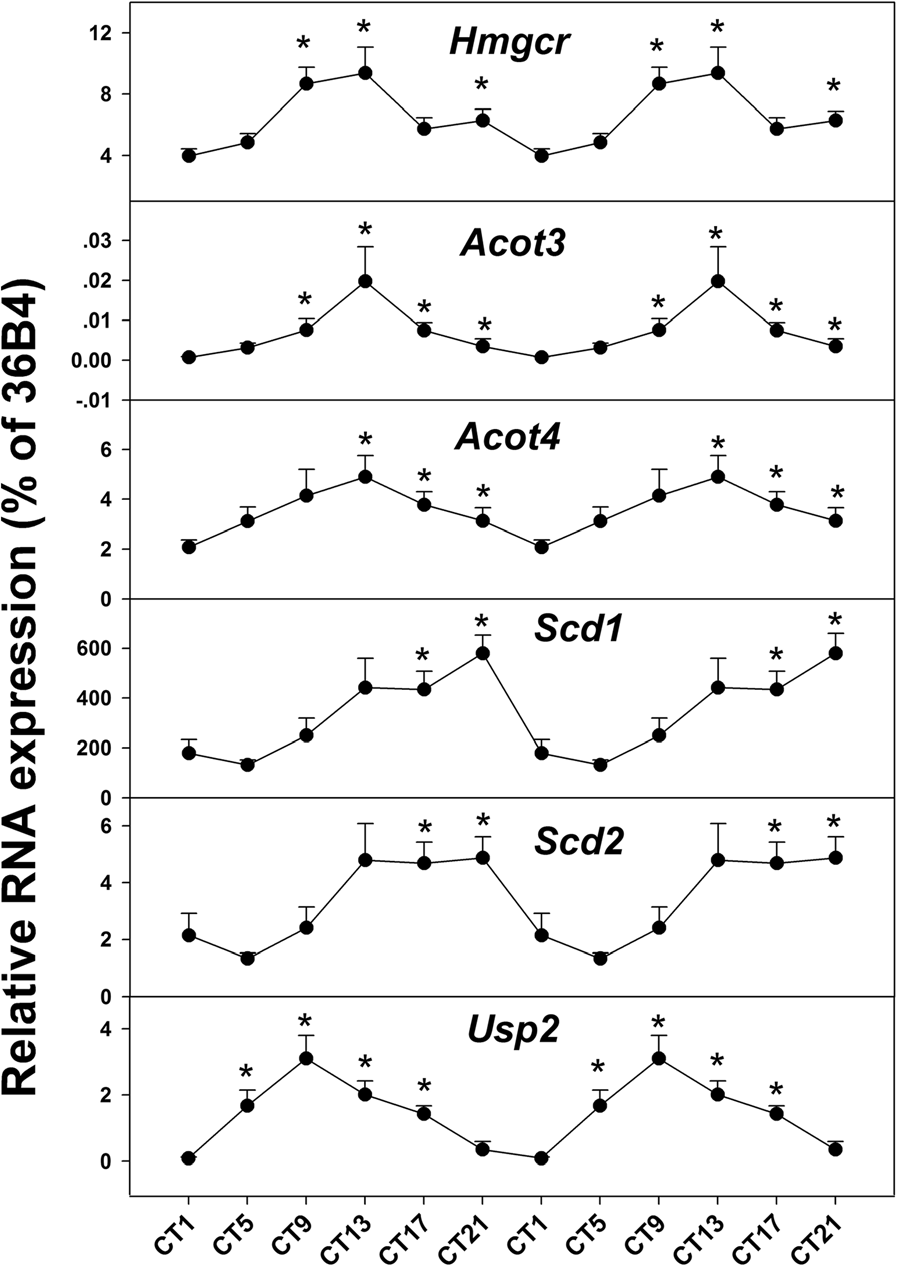
RT-qPCR analysis of selected genes in the L-DD model. Mice were acclimated to constant dark for 6 weeks, and livers were harvested at 9:00 AM (CT1), and then every 4 h, (CT5, CT9, CT13, CT17, and CT21) for 6 time-points

It has been reported that several factors influenced the identification of RNA-seq oscillation genes, including: (1) number of time points and replicates, (2) choice of analysis algorithm, (3) method of read-depth normalization, (4) number of reads per sample, and (5) choice of statistical analyses. It should be noted that the computational approach is an expedient to generate synthetic test data, rather than an approach to identify bona fide cycling transcripts [41]. In order to verify the results of MetaCycle, RT-qPCR was performed on selected genes of our interest (Fig. 8). The expression of *Acot3* and *Acot4* oscillated rhythmically at 24 h cycle of mouse liver. Both highest expressions occurred at CT13, the amplitudes were 2.5 and 5 for *Acot3* and *Acot4*. *Scd1* and *Scd2* also exhibited robust rhythm. The expression of *Scd1* peaked at CT21 and nadir at CT2. For *Scd2*, the highest expression occurred from CT13 to CT21 and the lowest expression at CT2. Besides, *Hmgcr* and *Usp2* also displayed robustly oscillation and their expression peaked at CT13 and CT9 respectively.

## Discussion

The present study extended our recent publication [27], and screened the oscillation gene in mouse liver under L-DD through MetaCycle. Approximatively 12.1% of the genes exhibited 24 h oscillation under L-DD. The KEGG and GO analysis further revealed circadian significance of oscillated genes. The oscillation genes under L-DD were enriched in the circadian rhythm pathway. The endogenous circadian rhythm of clock genes, P450 genes and lipid metabolism genes under L-DD were further compared with LD and S-DD. The oscillation patterns were similar but the period and amplitude of those oscillation genes were slightly altered. This is the first study to profile CT gene expressions under L-DD, indicating that the circadian rhythm of clock genes, P450 genes and lipid metabolism genes still robustly persists under L- DD. Light was not the necessary factor for persisting circadian rhythm but influenced the period and amplitude of oscillation of metabolism genes.

Consistent with the literature where animal circadian rhythms persist under constant darkness [42], the present study revealed that when mice are housed in constant darkness for up to 6 weeks, hepatic circadian rhythm still persists. Oscillation genes under LD, S-DD and L-DD were enriched in different biological process respectively, which was caused by an altered light cycle. As shown in Venn Diagram, more than 60% of oscillation genes kept rhythmicity only in their unique light cycle, once the light cycle changed, their oscillation disappeared. For example, circadian rhythm of reactive oxygen species (ROS) levels in *Daphnia pulex* are changed in different light condition [43]. The mRNA expression of Connexin30 and Connexin43 in mouse SCN exhibit oscillation under LD condition but disappeared in DD and LL [44]. Therefore, the oscillation of some genes depends on the entrainment of light while the others are endogenous.

For circadian clock genes, they displayed robust rhythm under L-DD conditions. Their oscillations were endogenous and knock out of these genes resulted in the loss of rhythms. For example, under DD conditions for 8 days, all wild-type mice remain rhythmic, but 2/3 of *Clock* mutant mice are arrhythmic [45]. Mice deficient in both mPer1 and mPer2 do not express circadian rhythms [46]. In 2-weeks DD conditions, *Rev-erbα* and *Rev-erbβ* retain dynamic oscillation throughout the 24-h cycle both in the SCN and liver [19], and when liver-specific knocks out *Rev-erbα* and *Rev-erbβ* genes, the circadian rhythm of *bmal1*, *clock* and output genes are all lost [47]. Therefore, light cycle is not necessary for endogenous circadian rhythm but influences the period and amplitude of oscillation genes. As shown in Table 1, L-DD lengthened the cycle of most circadian clock genes. Lack of light entrainment could explain why the period of most circadian clock genes became longer under L-DD.

In the present study, the P450 superfamily genes also exhibited robust circadian rhythm under DD conditions, and the peak of those oscillation genes occurred at the time of transition from light to dark. P450 genes are involved in metabolism of drug, xenobiotics, steroids and fatty acids [30]. It has been reported that circadian oscillators orchestrate the circadian rhythm of CYPs, for example, *DBP* regulates the circadian expression of CYP2A, 7A [48]. *RORα/γ* regulates the rhythm of CYP2B, 4A [49, 50]. CREM modulates the circadian expression of *Cyp51* [35]. Thus, without entrainment of light, P450 genes still can keep oscillation under L-DD condition. Abnormal light influences xenobiotic metabolism and detoxification. Light signals induce transcription of heme oxygenase 2 and cytochrome P450 oxidoreductase [51]. Compared to LD conditions, mice under DD and LL reduce ethanol intake and ethanol preference [52]. Moreover, compared with LD condition recovery, constant darkness results in a faster recovery of both motor and anxiety impairments in alcohol hangover swiss mice [53]. Profiling genes circadian rhythm under L-DD is important for metabolic study.

Cholesterol and fatty acids metabolism are regulated by the circadian clock [11, 54]. The present study uncovered that some genes involved in cholesterol and lipid metabolism maintained rhythm under L-DD conditions (Fig. 7). The peak (nadir) of those oscillation genes under L-DD occurred at CT9 or CT13. The circadian rhythm of *Acot3* and *Acot4* are evidently induced by calorie restriction and are transcriptional targets of *PPARa* [55]. Compared to LD, the circadian rhythm of *Acot3*, 4, 8 and 13 were also induced by L-DD with increased amplitude. Besides, the RT-qPCR also illustrated the oscillation of *Acot3* and *Acot4*. It has been reported that *Lipin1* has effects on both lipid synthesis and fatty acid oxidation to maintain lipid metabolic homeostasis [38]. Revealing its circadian rhythm under LD, S-DD and L-DD would help to understand the paradoxical efforts on lipid metabolism. The important lipogenic enzymes *Scd1* and *Scd2* are regulated by *Bmal1* [56], and oscillate under LD and S-DD but not L-DD. However, RT-qPCR results illustrated that there were strong daily fluctuations in the expression of *Scd1* and *Scd2*. Our pervious study illustrated the circadian rhythm of *Angptl8* [27]. The presented study further revealed the circadian rhythm of *Angptl3* and *Angptl4*, which oscillated both under LD and L-DD. Abnormal light causes metabolic disorders. Our pervious study illustrated chronic exposure to green light aggravates high-fat diet-induced obesity and metabolic disorders in male mice [57]. L-DD is also thought to be a factor of light. Therefore, profiling circadian rhythm of cholesterol and lipid metabolism genes under L-DD would contribute to better understanding lipid metabolic homeostasis.

In Fig. 8, RT-qPCR was performed on genes of interest to further verify RNA sequence findings. *Hmgcr* is crucial in cholesterol metabolism and is the target of statin drugs to treat hypercholesterolemia [58]. Also, ACOTs constitute a family of enzymes that hydrolyze fatty acyl-CoAs to form FFA and CoA. Existing evidence suggests regulatory roles in controlling rates of peroxisomal and mitochondrial fatty acyl-CoA oxidation [37]. *Scds* are responsible for mitochondrial fatty acid β-oxidation of short chain fatty acids, and shift food consumption away from fat and toward carbohydrate, controlling lipid handling in the liver [39]. For these selected genes, RT-qPCR verified their rhythms and confirmed the MetaCycle results.

## Conclusions

In summary, the present gene profiling study revealed that under L-DD, endogenous circadian clock genes exhibited typical oscillation patterns, which drive the metabolic genes, including P450 genes and lipid metabolism genes in a diurnal variation pattern. Compared with LD and S-DD conditions, the oscillation patterns of these metabolism genes under L-DD were similar but the period and amplitude of those oscillation genes were slightly altered. These endogenous circadian rhythms are biological signals cross-talking with metabolism genes to maintain physiological functions.

## Methods

### Animal treatment

Six-week old SPF-grade male mice (C57BL/6 J) were purchased from Model Animal Research Center of Nanjing University (Nanjing, Jiangsu, China). The animal experiments were conducted according to the Guide for the Care and Use of Laboratory Animals published by the National Institutes of Health (NIH publication) and approved by the Laboratory Animal Care Committee at China Pharmaceutical University (Permit number SYXK-2018-0019). Mice were housed for 1-week acclimation under a 12:12-h light/dark cycle, controlled temperature (22–24 °C) and humidity (50–60%) with free access to water and standard rodent chaw (normal diets with 4% of energy from lipid, XieTong Organism, China).

Subsequently, 42 mice were randomly divided into six groups, and each group had 7 mice, with 3–4 mice per cage. The animals were kept in constant dark condition (DD) for 6 weeks to eliminate bright light influence before experiments. Mice were sacrificed by dislocation of the cervical vertebrae. Livers were collected starting from 9:00 AM (CT1), and then every 4 h (13:00; 17:00; 21:00; 1:00; 5:00), corresponding to CT5, CT9, CT13, CT17 and CT21.

### Transcriptome sequencing

The liver samples were homogenized, and total RNA was extracted using TRIzol reagent (Molecular Research Center, USA). RNA quality and quantity were determined with Agilent 2200 TapeStation (Agilent Technologies, USA), with 260/280 > 1.8, and 260/230 > 1.4 as a quality reference. Pooled samples from 7 mice at each time point were sent to Guangzhou RiboBio Co., Ltd. (Guangzhou, China) for RNA sequencing, using Illumina HiSeq3000. The cDNA libraries were constructed with the Illumina Sample Preparation protocol (Illumina, San Diego, CA) to generate 100-bp paired-end reads. The raw sequencing data (FASTQ files) was aligned to the mouse genome (NCB137/mm10) using Bowtie. The RNA-seq data under 6 weeks of constant darkness (called long time dark-dark condition, L-DD) was deposited, and the GEO number is GSE133342 [27]. To compare gene oscillation pattern under normal light-dark condition (LD) and short time (2 days) dark-dark condition (S-DD), the data were retried from GEO database for LD (GSE114400, [28]) and S-DD (GSE70497, [29]).

### Normalization and oscillation genes identification

To avoid the differences of sequencing depths from different libraries, the method TMM was used to normalize the three RNA-sequences through R (64-bit, version 3.6.1) package “EdgeR” [59]. After the normalization, MetaCycle, an R package that incorporates ARSER, JTK_CYCLE and Lomb-Scargle to conveniently evaluate periodicity in time-series data, was performed to identify oscillation genes [60]. The output result of MetaCycle contained *p*-value, period, and revised amplitude. Oscillating transcripts were defined as *P < 0.05*. Heatmaps were generated using the pheatmap package to display the oscillation genes. The Venn Diagram Plotter was used to compare the rhythmic genes under LD, S-DD and L-DD and generated by VennDiagram package [61].

### Gene ontology (GO) and Kyoto encyclopedia of genes and genomes (KEGG) analysis

To understand biological function of the rhythmic genes under LD, S-DD and L-DD, GO (Biological process) and KEGG pathways were performed using R package clusterProfiler [62]. A value of *P* < 0.05 was used as a cutoff for significantly enriched terms. The graphs were drawn by R package Enrichplot [63].

### RT-qPCR analysis

Total RNA was reverse transcribed to cDNA with TakaRa RT kits (Takara Bio, Japan) according to manufacture instructions. The 10 μL PCR reaction mix contained 2 μL of cDNA (10 ng/ml), 5 μL qPCR SYBR mix (Takara Bio, Japan), 0.3 μL of primer mix (25 μM each) and 2.7 μL of ddH_2_O. The Real-time qPCR cycler (Roche Light Cycler 480 II) program was 5 min at 95 °C, 40 cycles of annealing and extension at 60 °C for 30 s and denaturation at 95 °C for 10 s. Dissociation curve was performed after the 40 cycles are over to verify the quality of primers and amplification. The values obtained were used to calculate the expression of clock genes by the 2^−△△Ct^ method and normalized to the housekeeping gene *36B4*. The relative transcript levels were calculated setting the control as 100%.

### Statistical analysis

The RT-qPCR data was presented as group mean with SEM (*n* = 7). Statistical analysis was completed using Statistical Package for the Social Sciences (SPSS) software (version 17.0). Before statistical analysis, all the data were inserted into the software to guarantee they followed normal distributions. Student’s t test was used to compare the differences between CT1 and the other time points. *P* < 0.05 was considered to indicate a statistical significance.

## Supplementary information

**Additional file 1.** Oscillation genes under L-DD. RNA-Seq data of L-DD was subjected to MetaCycle to screen oscillation genes.

**Additional file 2.** Oscillation genes under LD. RNA-Seq data of LD was subjected to MetaCycle to screen oscillation genes.

**Additional file 3.** Oscillation genes under S-DD. RNA-Seq data of S-DD was subjected to MetaCycle to screen oscillation genes.

## Data Availability

Three sets of RNA-seq data (Accession number: GSE114400, GSE70497, GSE133342) generated and/or analyzed during the current study are available in the GEO repository, https://www.ncbi.nlm.nih.gov/geo/query/acc.cgi.

## Abbreviations

Acot3,4,8,13: Acyl-CoA thioesterase3,4,8,13
Angptl3,4: Angiopoietin like 3,4
CT: Circadian time
Cyps: Cytochrome P450 family genes
DD: Constant darkness
GO: Gene ontology
KEGG: Kyoto encyclopedia of genes and genomes
LL: Constant light
Scd1,Scd2: Stearoyl-Coenzyme A desaturase1, 2
SCN: Hypothalamic suprachiasmatic nucleus
TMM: Trimmed mean of M-values normalization method
ZT: Zeitgeber time
